# Co-expression analysis of differentially expressed genes in hepatitis C virus-induced hepatocellular carcinoma

**DOI:** 10.3892/mmr.2014.2695

**Published:** 2014-10-17

**Authors:** QINGFENG SONG, CHANG ZHAO, SHENGQIU OU, ZHIBIN MENG, PING KANG, LIWEI FAN, FENG QI, YILONG MA

**Affiliations:** Department of Interventional Radiology, Affiliated Tumor Hospital of Guangxi Medical University, Nanning, Guangxi 530021, P.R. China

**Keywords:** hepatocellular carcinoma, hepatitis C virus, differentially expressed genes, time series genes, co-expression networks

## Abstract

The aim of the current study was to investigate the molecular mechanisms underlying hepatitis C virus (HCV)-induced hepatocellular carcinoma (HCC) using the expression profiles of HCV-infected Huh7 cells at different time points. The differentially expressed genes (DEGs) were identified with the Samr package in R software once the data were normalized. Functional and pathway enrichment analysis of the identified DEGs was also performed. Subsequently, MCODE in Cytoscape software was applied to conduct module analysis of the constructed co-expression networks. A total of 1,100 DEGs were identified between the HCV-infected and control samples at 12, 18, 24 and 48 h post-infection. DEGs at 24 and 48 h were involved in the same signaling pathways and biological processes, including sterol biosynthetic processes and tRNA amino-acylation. There were 22 time series genes which were clustered into 3 expression patterns, and the demarcation point of the 2 expression patterns that 401 overlapping DEGs at 24 and 48 h clustered into was 24 h post-infection. tRNA synthesis-related biological processes emerged at 24 and 48 h. Replication and assembly of HCV in HCV-infected Huh7 cells occurred mainly at 24 h post-infection. In view of this, the screened time series genes have the potential to become candidate target molecules for monitoring, diagnosing and treating HCV-induced HCC.

## Introduction

Hepatitis C virus (HCV) is characterized histologically by a persistent immune and inflammatory response that fails to clear HCV from hepatocytes (1). HCV is a leading cause of chronic liver disease and affects ~170 million people worldwide (2). Infection with HCV is a major challenge to public health (3); in the majority (50–80%) of infected individuals, the infection leads to chronic hepatitis, which progressively develops into hepatosteatosis, liver fibrosis, liver cirrhosis and ultimately to hepatocellular carcinoma (HCC) (4,5).

Genome-wide association studies have suggested that the natural course of HCV infection might be affected by the genetic background of the host (6,7). Thus, characteristics of the host and of the virus are have been widely studied and are considered to affect the process of hepatocarcinogenesis in a complex manner. A previous study has suggested that HCV-induced cellular responses may contribute to chronic liver diseases in various ways, including modulation of cell proliferation, alteration of lipid metabolism, and potentiation of oncogenic pathways (8). Smith *et al* (9) determined a HCC marker set that contained several cancer-related genes, including serine/threonine kinase 15 (STK15), plasma glutamate carboxypeptidase (PGCP) and two secreted A2 phospholipases (PLA2G13 and PLA2G7). These may provide potential HCC serological markers as they were strongly upregulated in more than half of the tumors analyzed by Smith *et al* (9). Furthermore, the HCV E2 protein activates the mitogen-activated protein kinase (MAPK) pathway in human hepatoma Huh7 cells and promotes cell proliferation (10,11). In human HCC, the expression levels of Spred protein, an inhibitor of the Ras/Raf-1/extracellular signal-related kinases (ERK) pathway, are deregulated (11,12). Therefore, there is an association between MAPK-ERK pathway activation and HCC.

The current therapy against HCV infection is limited by multiple factors, including resistance, adverse effects and high costs (13). Although the clinical development of novel antivirals targeting HCV protein processing has been shown to improve sustained virological response, toxicity of the individual compounds and development of viral resistance remain major challenges (14,15). Therefore, there is a requirement for studies concerning the molecular mechanism underlying HCV infection in the pathogenesis of chronic liver diseases and for the development of novel antiviral preventive and therapeutic strategies.

In the current study, changes in expression levels of a number of HCV infection-related genes in Huh7 cells at different time points were analyzed. The differentially expressed genes (DEGs) were identified with the Samr package in R software once the data were normalized. In addition, functional and pathway enrichment analysis of the identified DEGs was performed. Subsequently, MCODE in Cytoscape software was applied to conduct module analysis of the constructed co-expression networks. Results of the current study may aid the discovery of novel therapeutic targets and the development of new intervention strategies in HCV-mediated HCC.

## Materials and methods

### Samples

The gene expression profile of GSE20948 (16) was downloaded from the National Center for Biotechnology Information Gene Expression Omnibus (http://www.ncbi.nlm.nih.gov/geo/). A total of 28 specimens at 5 time points were available for analysis. At 6 h post-infection, there were only 2 repeated HCV-infected Huh7 cell specimens and 2 control specimens untreated with HCV, while at other time points (12, 18, 24 and 48 h post-infection), there were 3 repeated HCV-infected Huh7 cell specimens and 3 control specimens.

### Data preprocessing

The Robust Multi-array Average algorithm from the Affy package (Affymetrix, Santa Clara, CA, USA) in R statistical software (http://cran.at.r-project.org/) was employed to convert probe-level data in CEL files into expression measures (17). The probe numbers were then converted into gene names by combining annotation resources of R/Bioconductor (http://bioconductor.org/) (18) with the chip platform, which was provided by the Human Genome U133 Plus 2.0 Array (Affymetrix, Inc., Santa Clara, CA, USA). For each sample, the expression profile data were standardized by taking the mean expression values.

### Identification of DEGs

The Samr package (http://cran.r-project.org/web/packages/samr/index.html) in R software was employed to identify DEGs between HCV-infected and control samples at the 12, 18, 24 and 48 h time points (19). The 6 h time point data were excluded as there were only 2 repeated samples at this time point. The Δ value = 1, minimum fold change = 1 and a false discovery rate (FDR) <0.05 were used as the cut-off criteria. Fold change (fold change = expression value of HCV-infected sample/expression value of control sample) was calculated and defined as an expression value of each gene at each time point.

### Function and pathway enrichment analysis

The Database for Annotation, Visualization and Integrated Discovery (DAVID) (20) is a high-throughput and integrated data-mining environment that analyzes gene lists derived from high-throughput genomic experiments (21). In the present study, DAVID was utilized to conduct Gene Ontology (GO) function and Kyoto Encyclopedia of Genes and Genomes (KEGG) pathways enrichment analysis of DEGs, and to identify significantly enriched GO terms and KEGG signaling pathways (FDR <0.05). Overlapping DEGs at 12, 18, 24 and 48 h post-infection were identified and compiled to create a corresponding Venn diagram. The DEGs that occurred at these 4 time points were defined as time series genes. Overlapping DEGs were used for clustering analysis. It has previously been demonstrated that there is an incubation period after HCV infection (22), so the present study focused on DEGs at the 24 and 48 h time points post-HCV infection.

### Co-expression network construction

Genes that have similar functions or operate in the same pathway exhibit similar expression patterns when they are at the same physiological or time node (23). Construction of gene co-expression networks will contribute to the identification of gene sets of specific signaling pathways or biological processes. The fold change of each DEG was used as the expression value of the specific time point. Pearson correlation coefficients of any 2 genes were calculated. Gene pairs with a threshold value <0.95 were selected to build co-expression networks. Corresponding DEGs at 4 time points (12, 18, 24 and 48 h post-infection) were screened and 4 gene co-expression networks were constructed.

### Module identification of DEG co-expression networks

MCODE in Cytoscape software (http://www.cytoscape.org/) was applied to excavate functional modules (24). MCODE arithmetic consists of three stages: i) Vertex weighting; ii) complex prediction; and iii) post processing, in which the nodes of the predicted complex are filtered out or added based on connection indicators. To identify a highly interconnected or dense region of the network, the MCODE used a vertex-weighting scheme based on the clustering coefficient, Ci [Ci = 2^*^n/Ki^*^(Ki-1)]. Ki represents the node count of the neighborhood of node i; and n represents the number of edges among the Ki nodes in the neighborhood. The highest weighted vertex is a center point, seed of the region and search node j whose weight ratio (Wj/Wseed) was >0.1. It filters the predicted complexes if the minimum degree of the graph is less than the threshold, then constructs a module. The searched node is deleted from the network. The top 5 modules with a node count >10 in the co-expression networks were selected for functional enrichment analysis.

## Results

### Data preprocessing and identification of DEGs

For database GSE20948, a total of 19,944 gene expression values were obtained from 28 samples after data preprocessing. A total of 1,100 DEGs were identified between HCV-infected and control samples at 12, 18, 24 and 48 h post-infection. The number of DEGs at each time point is shown in Table I. Based on the same screening criteria, the numbers of DEGs were increased as time passed. The number of DEGs at 24 h post-infection was ~5 times more than the number at 18 h post-infection.

### GO and pathway enrichment analysis

The significantly enriched GO terms and KEGG signaling pathways are shown in Table II. The functions of DEGs at 18 h post-infection were decentralized and not enriched in any specific signaling pathways or biological processes. By contrast, functions of DEGs at the other 3 time points, particularly at 24 and 48 h post-infection, were enriched in post-transcription processes, such as sterol biosynthetic processes and tRNA amino-acylation, involving alanyl-tRNA synthetase (AARS) and threonyl-tRNA synthetase. Furthermore, DEGs at 24 and 48 h time points were enriched in the same signaling pathways and biological processes.

### Expression changes of DEGs with time

**Figure 1:**
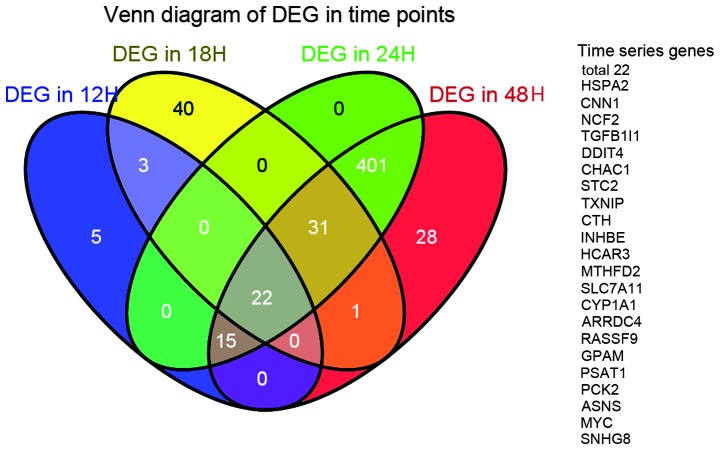
Overlapping of differentially expressed genes (DEGs) at different time points. The blue, yellow, green and red oval diagrams represent the DEGs at 12, 18, 24 and 48 h post-infection, respectively. The shadows of corresponding colors represent overlapping genes of different time points. A total of 22 DEGs were simultaneously presenting at 4 time points; 401 DEGs were simultaneously presenting at 24 and 48 h post-infection.

**Figure 2:**
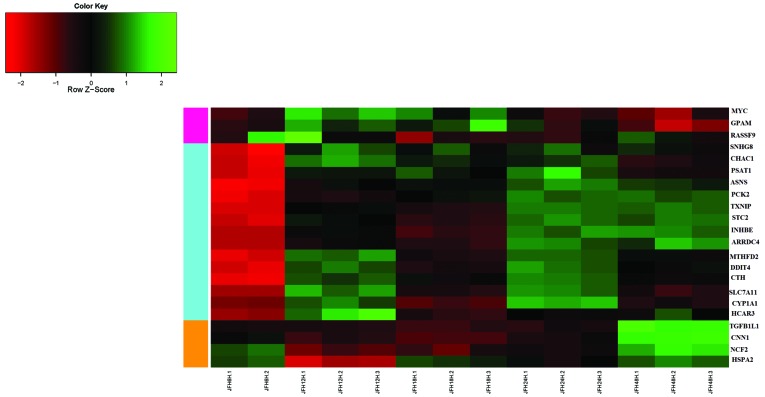
Expression patterns of time series genes with time. Horizontal axis represents time series samples at 6, 12, 18, 24 and 48 h. Right-hand vertical axis represents gene names. Left-hand vertical axis represents the clustering of time series genes, including 3 clustering patterns. Red represents the tendency of firstly increasing then decreasing; sky blue represents the tendency of firstly decreasing, then increasing and then stabilizing; yellow represents the tendency of first decreasing then increasing.

**Figure 3:**
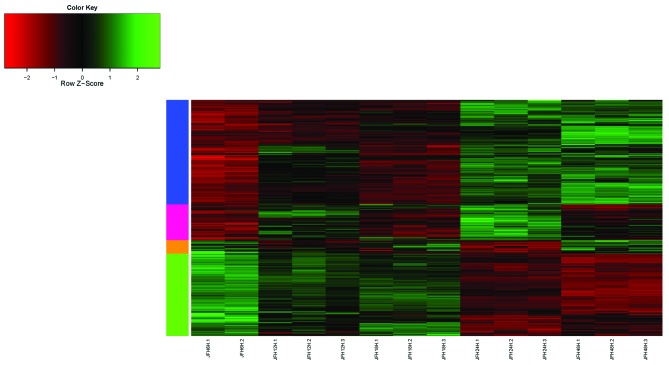
Expression patterns of overlapping differentially expressed genes (DEGs) at 24 and 48 h. Horizontal axis represents time series samples at 6, 12, 18, 24 and 48 h. Right-hand vertical axis represents gene names. Left-hand vertical axis represents the clustering situation of time series genes, including 2 clustering patterns. Blue and red indicate an initial decrease followed by an increase; yellow and green indicate an initial increase followed by a decrease. The demarcation point of the two expression patterns is 24 h.

In order to analyze the difference between DEGs at each time point, a Venn diagram was utilized to identify overlapping genes (Fig. 1). A total of 22 DEGs, denoted as time series genes, occurred in the HCV-infected samples at 12, 18, 24 and 48 h post-infection. To further study expression level changes of time series genes subsequent to HCV infection, the fold changes of these genes were clustered into a hierarchical diagram (Fig. 2). The 22 time series genes were clustered into three expression patterns. The expression levels of HSPA2, NCF2, CNN1 and TGFB1/1 first decreased and then increased. The expression levels of HCAR3, CYP1A1, SLC7A11, CTH, DDIT4, MTHFD2, ARRDC4, INHBE, STC2, TXNIP, PCK2, ASNS, PSAT1, CHAC1 and SNHG8 first decreased, then increased, and then stabilized. The expression levels of RASSF9, GPAM and MYC increased and then decreased. Furthermore, 401 DEGs were concurrent in Huh7 cells at 24 and 48 h post HCV-infection. These 401 DEGs were clustered into two expression patterns: i) Decreasing then increasing; and ii) increasing then decreasing. The demarcation point of the two expression patterns is at 24 h post-infection (Fig. 3). The functions of these DEGs at 24 and 48 h post HCV-infection are mainly related to cholesterol processing and amino-acyl tRNA synthetase signaling pathways (Table III).

### Module analysis of the constructed co-expression networks

**Figure 4:**
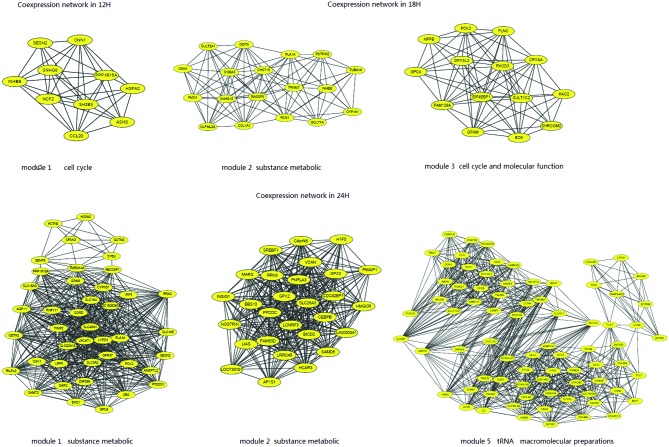
Specific functional modules of differentially expressed genes at 12, 18 and 24 h post-infection.

**Figure 5:**
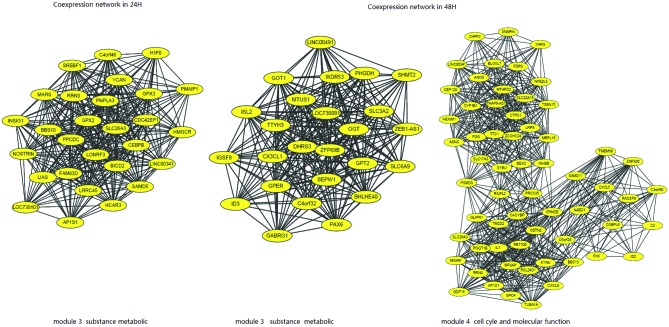
Specific functional modules of differentially expressed genes at 24 and 48 h post-infection.

All DEGs were the nodes of the co-expression networks. DEGs at 24 and 48 h presented high clustering and were more modular (Table IV). The top 5 modules with a node number >10 were screened out for GO functional analysis by DAVID. The functions of the significantly enriched modules included various biological processes (Figs. 4 and 5). The biological processes of cell proliferation and the cell cycle were decentralized, which suggested that HCV infection may influence the proliferation of host liver cells. The co-expression networks at 18, 24 and 48 h demonstrated that the metabolism and transport processes of multiple biological macromolecules, including amino acids, enzymes and carbohydrates, began to decrease. At 24 and 48 h, tRNA synthesis-related biological processes were emerging, suggesting that replication and assembly of HCV in HCV-infected Huh7 cells occurred mainly at 24 h post-infection.

## Discussion

HCV is a leading cause of chronic hepatitis, liver cirrhosis, and HCC in numerous areas of the world (25). A study has demonstrated that expression levels of host genes involved in cellular defense mechanisms, cellular metabolism and intracellular transport are significantly altered by HCV infection (26). Therefore, the identification and characterization of key host cellular factors that are involved in the HCV replication cycle are necessary for the understanding of disease pathogenesis and the identification of novel antiviral therapeutic targets.

In the present study, the changes in the expression levels of genes associated with HCV-infected hepatocyte regulation were examined using gene chips. A total of 1,100 DEGs were identified at 4 time points by the gene expression profiles of HCV-infected samples and control samples. The number of DEGs increased with the period of time after HCV infection. The significantly enriched GO terms and KEGG signaling pathways displayed that the functions of the DEGs were decentralized at 18 h time points, while the functions at the other 3 time points were focused on preparation of post-transcription, including activation of amino-acyl tRNA synthetase. Additionally, DEGs at 24 and 48 h were enriched in the same signaling pathways and biological processes. Amino-acyl tRNA synthetases are crucial for protein synthesis, and targeting the editing domain of this enzyme is a novel approach to its inhibition (27). HCV may interact with amino-acyl tRNA synthetases, which are the targets of several myositis-specific auto-antibodies (28). A study has demonstrated that amino-acyl tRNA synthetases are important in viral replication (29). Furthermore, AARS was involved in the amino-acyl tRNA synthetase pathway: Amino-acyl tRNA synthetases exhibit a vital role in protein synthesis by catalyzing the aminoacylation of tRNA with its cognate amino acid (30), thereby providing a central target for the decoding of genetic code during protein translation (31). A study suggested that AARS produce dinucleotide polyphosphates, which function as important signaling molecules (32). To further analyze DEGs at different time points, a Venn diagram was employed in the present study to explore the overlapping genes. The 22 DEGs, appearing at 4 time points after HCV infection, were defined as time series genes. The hierarchical diagram of these time series genes indicated that they presented 3 changing patterns. Additionally, the genes of HSPA2, TXNIP, CYP1A1 and GPAM were simultaneously enriched in GO terms and modules.

HSPA2 is known to interact with HIV-1 Gag to facilitate nuclear import of the viral preintegration complex (33). Neutrophil cytosolic factor 2 (NCF2) is the gene encoding the NADPH oxidase cytosolic component p67^phox^ (34).

Thioredoxin-interacting protein (TXNIP) is an α-arrestin family protein that is induced in response to glucose elevation (35). A previous study has shown that lack of TXNIP protects against diabetes and glucotoxicity-induced β-cell apoptosis (36). The knockdown of the TXNIP gene using small interfering RNA (siRNA) resulted in a reduction of up to 90% in HCV replication (37). This is a novel finding which may be of importance to the study of HCV, as TXNIP is involved in oxidative stress, lipid metabolism and glucose metabolism, all of which have the potential to influence the HCV life cycle. Furthermore, in another study it was determined that reducing the expression of host genes involved in lipid metabolism (TXNIP and CYP1A1 genes) and those involved in intracellular transport, reduced the replication and secretion of HCV, indicating that they may be important factors for the viral replication cycle (15). Study has been demonstrated that TXNIP deficiency impaired activation of the NLRP3 inflammasome and subsequent activation of IL-1β (38).

Cytochrome P450s are enzymes that catalyze Phase-1 metabolism reactions. Cytochrome P450 1A1 (CYP1A1) is a member of the CYP1 family and participates in the metabolism of a number of exogenous and endogenous substrates (39) The high epoxidation activities of CYP1A1, CYP2A6 and CYP3A4 were consistent with their pronounced susceptibilities to mechanism-based inactivation by chalepensin (40). The CYP1A1 gene has been implicated in the etiology of HCC (41). The knockdown of the genes ABLIM3, SPTLC3 and CYP1A1 using siRNA resulted in significant impairment of HCV replication (42). Molecules involved in lipid metabolism including TXNIP, CYP1A1 and CIDEC have been demonstrated to be essential for effective HCV replication and secretion (43).

Glycerol-3-phosphate acyltransferase (GPAM) is a key enzyme in the lipid biosynthesis of triacylglycerols and phospholipids. It was demonstrated that GPAM protein expression levels are linked to metabolomic and lipidomic profiles in a cohort study of human breast carcinomas (44). In the Huh7 cell line, miR-127 regulated the expression of several key lipid-metabolism genes, including GPAM and Angptl3 (45).

In the present study, GO functional analysis displayed that these DEGs were mainly enriched in cholesterol processing. Previously it has been suggested that the synthesis of fatty acids (FA) and cholesterol is dysregulated in HCV (46). A study demonstrated that FA and cholesterol are implicated in HCV progression (47). *De novo* lipogenesis may contribute to HCV-induced damage by involvement in the viral replication process, and by contributing FAs to lipid storage in the liver, leading to steatosis (48). Lipogenesis is elevated while cholesterol synthesis is impaired in HCV. The cellular Niemann-Pick C1-like 1 (NPC1L1) cholesterol uptake receptor is an HCV entry factor potentially applicable to therapeutic intervention (49,50). Another study demonstrated that the cholesterol and sphingolipids associated with HCV particles are important for virion maturation and infectivity, and that cholesterol depletion or hydrolysis of sphingolipids from HCV particles results in a loss of infectivity (51).

The module analysis of the co-expression networks exhibited that the biological processes of cell proliferation and the cell cycle were decentralized with time. The demarcation point of the changing patterns is 24 h. The overlapping genes of 24 and 48 h were mainly involved in cholesterol processing, including CCHC-type zinc finger (CNBP). CNBP is one of the most abundant genes in the retinal pigment epithelium (52). It is also a multifunctional nucleic acid chaperone involved in cell death and proliferation control (53). This implies that HCV infection affects cell proliferation of host liver cells. Also, HCV replication and assembling mainly occurred at 24 h post infection in the present study, which was consistent with the previous result of functional analysis of DEGs. Consistent with the previous result of functional analysis of DEGs, the DEGs at 24 and 48 h were also enriched in cholesterol processing and tRNA aminoacylation.

Overall, in the current study, the screened featured genes were clearly associated with amino-acyl tRNA synthetase following HCV infection. A comprehensive understanding of cancer progression may elucidate the genetic and molecular mechanisms of oncogenesis, and provide important information for effective diagnosis and prognosis of the disease. In addition, the findings have a great significance in the study of these mechanisms, and in probing normal samples and distinguishing them from cancerous samples. Regardless, further experiments are required to confirm the results of the present study.

## Figures and Tables

**Table I:** Number of DEGs at 12, 18, 24 and 48 h post-infection.

	Significantly regulated genes (n)		
	
Time (h)	Upregulated genes	Downregulated genes	Total
12	6	39	45
18	51	46	97
24	181	289	470
48	198	300	498

DEGs, differentially expressed genes.

**Table II:** Significantly enriched GO terms and KEGG signaling pathways of DEGs at different time points.

Time (h)	GO term and KEGG pathway	Count	FDR
12	GO:0006366~transcription from RNA polymerase II promoter	7	0.046289
24	GO:0016126~sterol biosynthetic process	9	0.002755
	GO:0006695~cholesterol biosynthetic process	8	0.003824
	GO:0008203~cholesterol metabolic process	13	0.004216
	GO:0016125~sterol metabolic process	13	0.011369
	GO:0043038~amino acid activation	9	0.024144
	GO:0006418~tRNA aminoacylation for protein translation	9	0.024144
	GO:0043039~tRNA aminoacylation	9	0.024144
	hsa00970:Aminoacyl-tRNA biosynthesis	10	0.004451
48	GO:0016126~sterol biosynthetic process	9	0.004102
	GO:0006695~cholesterol biosynthetic process	8	0.005448
	GO:0008203~cholesterol metabolic process	13	0.007293
	GO:0016125~sterol metabolic process	13	0.019449
	GO:0006418~tRNA aminoacylation for protein translation	9	0.035474
	GO:0043039~tRNA aminoacylation	9	0.035474
	GO:0043038~amino acid activation	9	0.035474
	hsa00970:Aminoacyl-tRNA biosynthesis	10	0.007020

GO, gene ontology; KEGG, Kyoto Encyclopedia of Genes and Genomes; DEGs, differentially expressed genes.

**Table III:** Enriched GO terms and KEGG pathways of overlapping DEGs at 24 and 48 h.

GO term and KEGG pathway	Count	Genes	FDR
GO:0008203~cholesterol metabolic process	13	SREBF1, CNBP, MVD, HMGCR, FDPS, HMGCS1, FDFT1, SQLE, DHCR7, INSIG1, CAT, VLDLR, NSDHL	0.00081
GO:0016126~sterol biosynthetic process	9	CNBP, MVD, HMGCR, SQLE, DHCR7, HMGCS1, FDPS, FDFT1, NSDHL	0.000844
GO:0006695~cholesterol biosynthetic process	8	CNBP, MVD, HMGCR, DHCR7, HMGCS1, FDPS, FDFT1, NSDHL	0.001338
GO:0016125~sterol metabolic process	13	SREBF1, CNBP, MVD, HMGCR, FDPS, HMGCS1, FDFT1, SQLE, DHCR7, INSIG1, CAT, VLDLR, NSDHL	0.002251
hsa00970:Aminoacyl-tRNA biosynthetic process	9	WARS, TARS, YARS, CARS, AARS, GARS, SEPSECS, MARS2, MARS	0.011377

GO, gene ontology; KEGG, Kyoto Encyclopedia of Genes and Genomes; DEGs, differentially expressed genes.

**Table IV:** Co-expression networks of DEGs at 12, 18, 24 and 48 h post-infection.

Time (h)	Node	Edge	Module
12	45	108	7
18	97	467	22
24	469	5676	23
48	498	5522	22

DEGs, differentially expressed genes.
